# Intraventricular meningiomas frequently harbor NF2 mutations but lack common genetic alterations in TRAF7, AKT1, SMO, KLF4, PIK3CA, and TERT

**DOI:** 10.1186/s40478-019-0793-4

**Published:** 2019-08-30

**Authors:** Gerhard Jungwirth, Rolf Warta, Christopher Beynon, Felix Sahm, Andreas von Deimling, Andreas Unterberg, Christel Herold-Mende, Christine Jungk

**Affiliations:** 1 Division of Experimental Neurosurgery, Department of Neurosurgery, University of Heidelberg, INF 400, D-69120 Heidelberg, Germany; 2 Department of Neuropathology, Institute of Pathology, University of Heidelberg, INF 224, D-69120 Heidelberg, Germany; 3 Clinical Cooperation Unit Neuropathology, German Consortium for Translational Cancer Research (DKTK), German Cancer Research Center (DKFZ), Heidelberg, Germany

**Keywords:** Intraventricular meningioma, NF2, Targeted panel sequencing, SMARCB1, SMARCA4

## Abstract

Intraventricular meningiomas (IVMs) account for less than 5% of all intracranial meningiomas; hence their molecular phenotype remains unknown. In this study, we were interested whether genetic alterations in IVMs differ from meningiomas in other locations and analyzed our institutional series with respect to clinical and molecular characteristics. A total of 25 patients with surgical removal of an IVM at our department between 1986 and 2018 were identified from our institutional database. Median progression-free survival (PFS) was 79 months (range of 2–319 months) and PFS at 5 years was 86%. Corresponding tumor tissue was available for 18 patients including one matching recurrence and was subjected to targeted panel sequencing of 130 selected genes frequently mutated in brain cancers by applying a custom hybrid capture approach on a NextSeq500 instrument. Loss of chromosome 22q and 1p occurred frequently in 89 and 44% of cases. Deleterious NF2 mutations were found in 44% of IVMs (*n* = 8/18). In non-NF2-mutated IVMs, previously reported genetic alterations including TRAF7, AKT1, SMO, KLF4, PIK3CA, and TERT were lacking, suggesting alternative genes in the pathogenesis of non-NF2 IVMs. In silico analysis revealed possible damaging mutations of APC, GABRA6, GSE1, KDR, and two SMO missense mutations differing from previously reported ones. Interestingly, all WHO°II IVMs (*n* = 3) harbored SMARCB1 and SMARCA4 mutations, indicating a role of the SWI/SNF chromatin remodeling complex in aggressive IVMs.

**Electronic supplementary material:**

The online version of this article (10.1186/s40478-019-0793-4) contains supplementary material, which is available to authorized users.

## Introduction

Intraventricular meningiomas (IVMs) are rare tumors; they account for only 0.5–5% of all intracranial meningiomas and up to 14% of all intraventricular tumors [5, 12, 19, 26, 35]. The most common tumor location is within the lateral ventricles (80%), whereas 15% of the IVMs arise from the third ventricle and 5% are located in the fourth ventricle [12, 13, 19]. They are thought to arise either from the choroid plexus or the tela choroidea within the ventricular system [35]. Surgical excision is considered the treatment of choice, but remains challenging due to deep tumor location, the presence of eloquent structures adjacent to the ventricles, and mostly large tumor size at diagnosis [19, 41]. The patients’ presenting symptoms depend on the tumor location, but include headache, hydrocephalus, visual impairment, and motor or sensory deficits [5, 13, 19, 35].

In meningiomas, there is increasing knowledge about their molecular phenotype. The most common genetic alteration found in meningiomas involves the tumor suppressor neurofibromatosis gene 2 (NF2) on chromosome (chr) 22q. Loss of heterozygosity (LOH) at this chromosomal region is typically detected in 40–80% of sporadic meningiomas [28, 31, 34]. Moreover, inactivating NF2 mutations can be found in up to 60% of the tumors, supporting the classical two-hit hypothesis in meningioma pathogenesis [45]. Genetic alterations of NF2 include insertions, deletions, nonsense mutations or affecting splice sites, producing a truncated, non-functional protein [30]. Despite genetic alterations of the NF2 gene, recent studies revealed other important alterations in non-NF2 meningiomas. The second most frequently mutated gene in meningiomas involves the tumor suppressor TNF receptor associated factor 7 (TRAF7) gene [9]. This alteration is highly associated with AKT Serine/Threonine Kinase 1 (AKT1 E17K), Krueppel-like-factor 4 (KLF4 K409Q) or Phosphatidylinositol-4,5-Bisphosphate 3-Kinase Catalytic Subunit Alpha (PIK3CA) mutations [1, 9]. Less frequently, yet completely independent of NF2/TRAF7 alterations, two recurring smoothened (SMO)-activating mutations were reported. SMO L412F and SMO W535L resulted in an overexpression of the sonic hedgehog pathway [9]. Recently, telomerase reverse transcriptase (TERT) mutations in the promoter region were discovered in meningiomas. They were present in approximately 6% and were found to be associated with higher meningioma grades and early recurrence [46, 49]. Several members of the Switch/Sucrose Non-Fermentable (SWI/SNF) chromatin remodeling complex have been implicated in meningioma pathogenesis. Somatic SWI/SNF-related matrix-associated actin-dependent regulation of chromatin subfamily B member 1 protein (SMARCB1) mutations have been identified in rare spontaneous meningiomas [6, 20].

Recent studies revealed the association of meningioma location and distinct mutations. For instance, NF2/chr22loss meningioma meningiomas typically originate from the lateral regions and posterior fossa, while the vast majority of non-NF2 meningiomas commonly harbor location-specific mutations in SMO (olfactory groove), KLF4/TRAF7 (medial skull base), and AKT1/TRAF7 (anterior skull base) [1, 6, 9, 20]. However, no molecular data exist on molecular alterations in IVMs. Hence, we were interested whether genetic alterations in IVMs differ from meningiomas in other locations and investigated our institutional series on a molecular level.

## Materials and methods

### Clinical data, tumor samples and DNA isolation

Our institutional database was screened for patients with surgical resection of an IVM at the Department of Neurosurgery at the University Hospital Heidelberg, Germany between 1986 and 2018. Demographic, tumor-related (tumor location, tumor size, WHO grade, histological subtype), treatment-related and outcome data were collected retrospectively from medical charts’ review and magnetic resonance imaging (MRI) studies. Tumor size was calculated by MRI scans with the formula *Volume = (Width^2 x Length)/2*. Progression-free survival (PFS) was defined as the interval from 1st surgery until last MRI scan while follow-up was defined as the interval from 1st surgery until last patient contact. The extent of resection (EOR) was determined on the basis of surgical reports or early postoperative MRI scans if available. EOR was categorized in gross total resection (GTR, Simpson °I-III) or subtotal resection (STR; Simpson °IV-V) according to Simpson’s grading. Fresh tumor material obtained intraoperatively was immediately snap-frozen and stored at − 80 °C until further processing. Histological diagnosis and grading of IVMs was based on the World Health Organization Classification of Central Nervous System Tumors in use at the time of 1st surgery. Only tissue samples with a vital tumor cell content > 60% as determined on hematoxylin and eosin stained slides by a board-certified neuropathologist (Department of Neuropathology, University Hospital Heidelberg, Germany) were processed further. DNA was extracted from tumor tissues using the AllPrep Kit (QIAGEN, Venlo, Netherlands) according to the manufacturer’s instructions. DNA amount was quantified by NanoDrop ND-1000 spectrophotometer (Thermo-Scientific, Waltham, MA, USA) and then stored at − 80 °C until further analysis. The institutional review board at Heidelberg Medical Faculty approved this study. Written informed consent was retrieved from all patients.

### Targeted panel sequencing

IVM tissue samples were profiled by targeted panel sequencing. The panel contained 130 genes reported to be frequently mutated in brain tumors including meningiomas as described previously [47]. The panel was designed to assess the frequency of known mutations and was not designed to detect novel mutational events. Notably, all mutations described in meningiomas at the time of the design of this panel are covered. Sequencing was done by applying a custom hybrid capture approach (Agilent Technologies, CA, USA) on a NextSeq500 instrument (Illumina, San Diego, CA, USA) with an average coverage of over 500-fold.

### Data processing

Raw data were processed as described before [47]. Briefly, raw data were de-multiplexed and converted into the fastq format with the “bcl2fastq” tool from Illumina. With the data in the standard fastq format, quality checks were performed with the tool fastqc. The resulting fastq files were processed with BWA mem to align the reads to the reference genome (currently GRch37 is used) [32]. From the generated bam-files, duplicated sequences were removed with picard tools, at which point the files are ready for further data processing. For single nucleotide variant calling, we used SAMtools MPileUp, while for InDel calling Platypus was utilized [25]. For further filtering to remove low quality calls the following filter steps are used (a) read depth greater or equal 40, genotype quality greater or equal 99, minimum allele frequency of 1%, at least 10% reads from each strand. Filtered results were then annotated by annovar [53] with additional information from databases such as dbSNP, 1000 Genomes Project and COSMIC as well as SIFT, PhyloP, PolyPhen2, LIB-LRT, and MutationTaster. Due to a lack of matched germline controls, data was compared with publicly available germline databases such as dbSNP, 1000 Genomes Project as described before [21]. Copy number variation plots were generated with the R bioconductor package seqCNA [36]. Automatic scoring for the detection of chromosomal gains and losses was verified by manual assessment of the respective loci for each individual profile as described [48].

### In silico prediction of possible pathogenic mutations

To identify novel, not previously reported mutations in IVMs, we used previously published in silico algorithms including SIFT, PhyloP, PolyPhen2, LIB-LRT, and MutationTaster to predict the impact of missense mutations on the protein function [2, 56]. As purely sequence-based prediction tool, SIFT classifies non-synonymous single nucleotide polymorphisms on the basis of the evolutionary conversation of amino acids within protein families. MutationTaster uses evolutionary conservation and splice site prediction, whereas PhyloP is solely based on DNA sequence conservation. PolyPhen-2 incorporates sequence-based and structural features as input if the 3D structure of the target protein is known. LRT uses a sequence evolutionary model to calculate the probability of possible damaging mutations. Due to the specific weaknesses of each prediction tool, a common strategy is to combine results of various approaches [15, 16]. Therefore, only genes were included for which all five algorithms resulted in the prediction of possibly damaging mutations.

## Results

### Tumor characteristics and clinical outcome of IVM patients

**Fig. 1:**
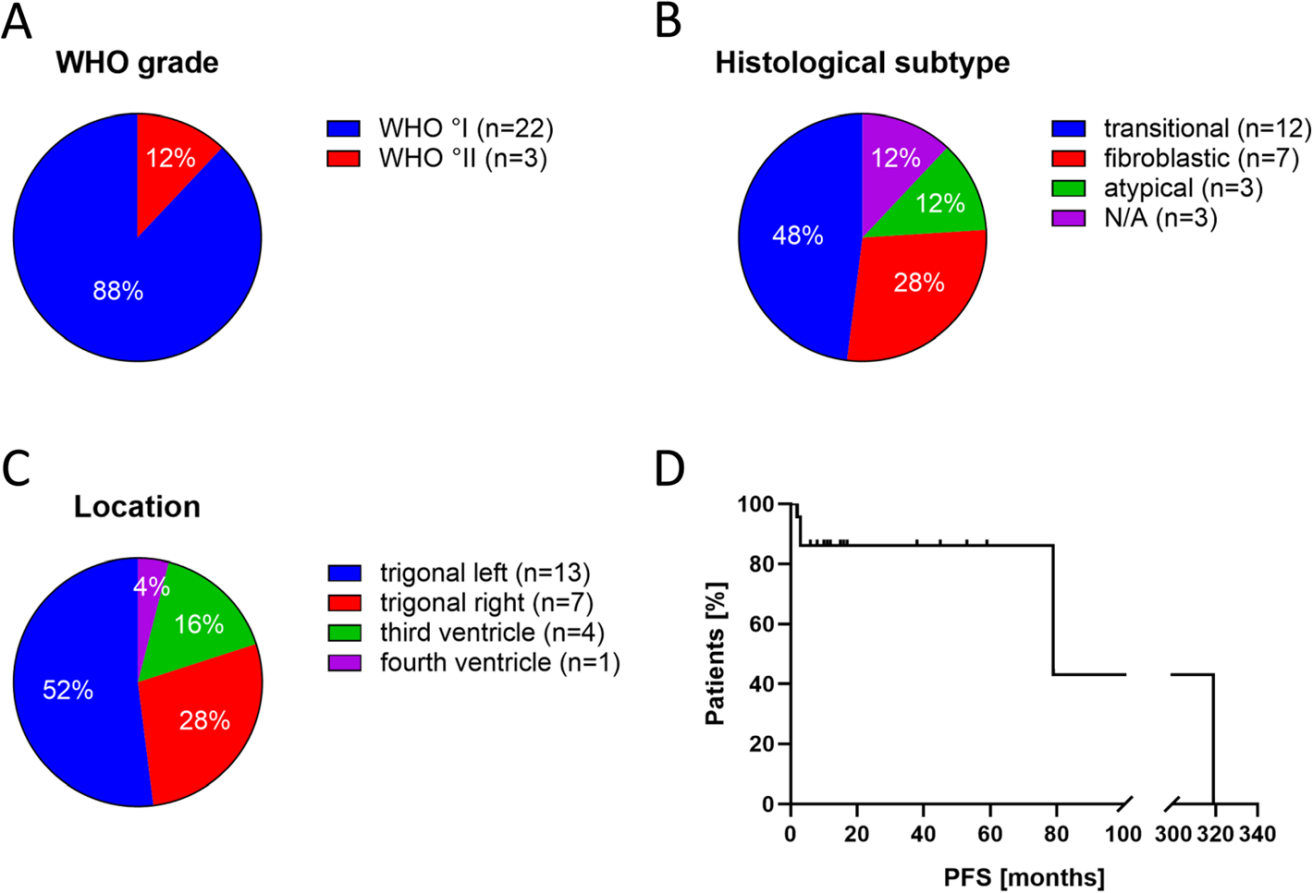
Clinical and demographic data of patients with intraventricular meningiomas (*n* = 25). Distribution of WHO grade (**a**), histological subtype (**b**) and location (**c**) among our series of 25 patients with IVMs. Progression-free survival (PFS) was determined on follow-up MRI scans (**d**)

From a consecutive cohort of more than 2200 meningiomas treated at our department between 1986 and 2018, we identified 25 patients diagnosed with IVM (1.1%) (Table 1). The median age at diagnosis was 58 years (range 14–75 years) with a female preponderance (female:male = 2.15:1). The patients’ presenting symptoms included headache (36.4%), psychoorganic syndrome (18.2%), motor deficits (13.6%), visual impairment (13.6%), papillary stasis (4.5%), and hydrocephalus (4.5%) (Table 2). In eight patients (36.4%), IVMs were incidental findings. 22 IVMs were graded as WHO°I (88%) and 3 IVMs as WHO°II (12%) (Fig. 1a, Table 1). Regarding histological subtypes, the transitional subtype was the most frequent one (*n* = 12, 48%), followed by the fibroblastic subtype encountered in 7 cases (28%) (Fig. 1b). The most common tumor location was within the lateral ventricles (80%) with the left lateral ventricle more frequently affected in 52% of all cases (*n* = 13) compared to the right lateral ventricle (38%, *n* = 7; Fig. 1c). In 4 cases (16%), tumors occurred within the third ventricle while only one IVM (4%) arose within the fourth ventricle. The median tumor size was 43 cm^3^, ranging from 0.8 to 134.6 cm^3^, calculated from preoperative contrast-enhanced T1-weighed MRI scans. GTR was achieved in 92% of the tumors (*n* = 23/25). Only one tumor underwent STR and in one IVM, the EOR was unknown. Two patients with meningioma WHO°II received postoperative radiotherapy. The median follow-up was 13 months (range 0.7 to 399 months). Meanwhile, radiographic tumor recurrence/progression was observed in five patients (*n* = 5/25, 20%). Median PFS was 79 months (range 2–319 months). PFS at 5 years among all tumors was 86% (Fig. 1d). In our series, no tumor-related death occurred.

**Table 1:** Clinical data of patients with intraventricular meningioma (*n* = 25)

clinical data	*n* = 25	[%]
Sex	[n]	
Male	8	32
Female	17	68
Age	[years]	
Mean	48	
Median	54	
Range	14–75	
WHO Grade	[n]	
WHO°I	22	88
WHO°II	3	12
Histological Subtype	[n]	
Fibroblastic	7	28
Transitional	12	48
Atypical	3	12
Mixed/Unknown	3	12
Location	[n]	
Trigonal left	13	52
Trigonal right	7	28
Third ventricle	4	16
Fourth ventricle	1	4
Tumor recurrence	[n]	
Recurrence	5	
Extent of Resection	[n]	
GTR (Simpson °I-°III)	23	92
STR (Simpson °IV-°V)	1	4
Unknown	1	4
Postoperative treatment	[n]	
Radiotherapy	2	
Chemotherapy	0	
Pre-operative tumor volume	[cm^3^]	
Median size	36.7	
Mean Size	43.0	
Range	0.8–134.6	
Follow-up	[months]	
Median	13	
Mean	50	
Range	0.7–399	
Progression free survival		
At 5 years	86%	
Median [months]	79 (2–319)

**Table 2:** Presenting symptoms of patients with intraventricular meningioma

Presenting symptoms	[n]	[%]
Headache	8	36.4
Incidental finding	8	36.4
Psycho-organic syndrome	4	18.2
Motor deficit	3	13.6
Visual impairment	3	13.6
Papillary stasis	1	4.5
Hydrocephalus	1	4.5

### Analysis of copy number variations reveal recurrent monosomy of 22q and 1p

**Fig. 2:**
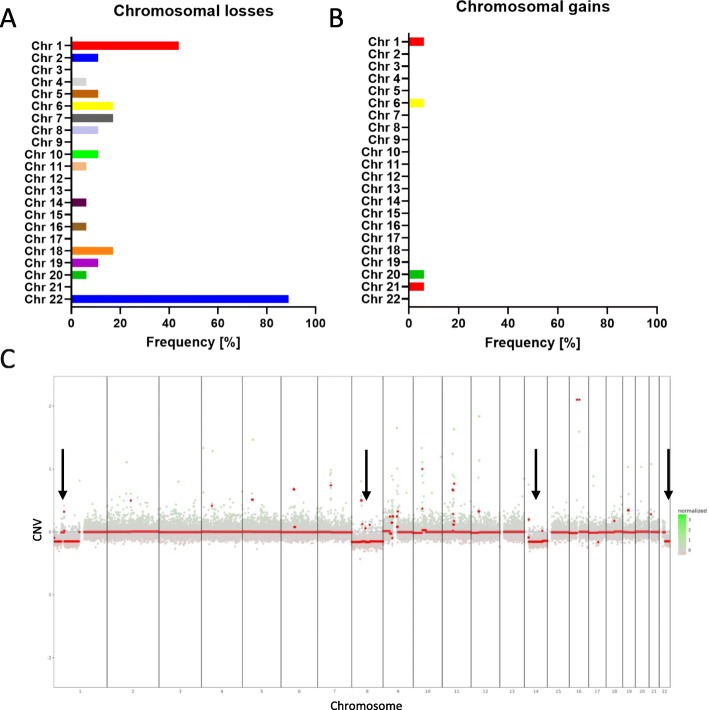
Chromosomal aberrations of intraventricular meningiomas (*n* = 18). Frequency of chromosomal losses (**a**) and gains (**b**) in intraventricular meningiomas assessed by copy number variants. Representative copy number plot derived from sequencing data with LOH of chr 1p, 8, 14 and 22q (**c**)

From our clinical cohort of 25 IVM patients, corresponding tumor samples were available for 18 patients including one matching recurrent WHO°II tumor (*n* = 15 WHO°I; n = 2 WHO°II, n = 1 recurrent WHO°II tumor) and were analyzed by targeted panel sequencing as described before [47]. This panel includes 130 of the most frequent gene mutations in brain tumors and covers all relevant gene mutations described in meningiomas. Chromosomal loss was most frequent (89%) at chromosome (chr) 22q, where the well-known tumor suppressor NF2 is located (Fig. 2a). Second most recurrent chromosomal alteration was loss of chr 1p in 44% of cases. Other chromosomal losses included chr 6/7/18 with a frequency of 17%, and all other alterations at chr 3, 12, 13, 17 and 21 affected single cases only. Gains were rarely detected in our IVM cohort (Fig. 2b). Only chr 1, 6, 20, and 21 showed gains in single cases. Figure 2c depicts a representative sample of chr 1p, 8, 14, and 22q loss. In summary, loss of chromosomes 22q and 1p seems to be a frequent event in IVMs.

### IVMs frequently harbor NF2 mutations and aggressive IVMs harbor SMARC mutations

**Fig. 3:**
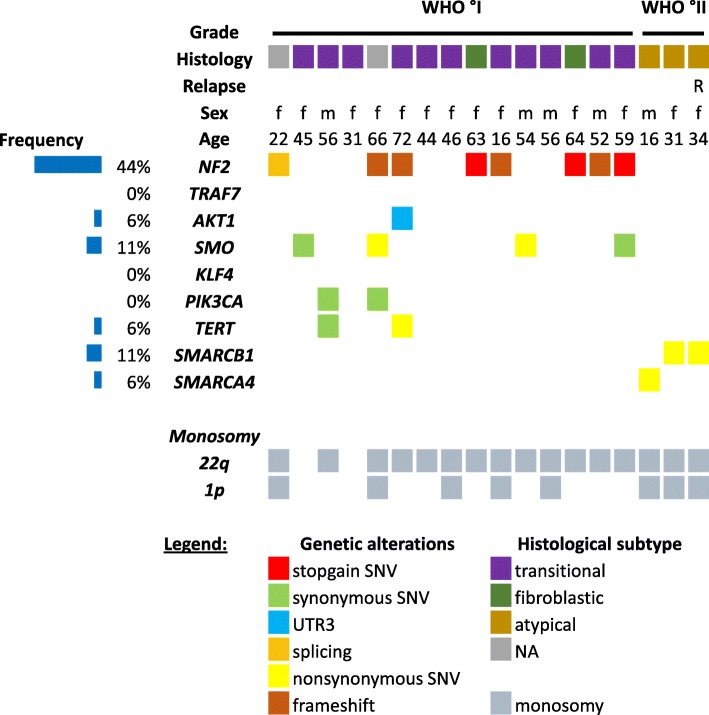
Common genetic alterations in NF2 and non-NF2 meningiomas (*n* = 18). Representation of WHO grade, histological subtype, sex, age and common genetic alterations found in meningiomas including NF2, TRAF7, AKT1, SMO, KLF4, PIK3CA, TERT, SMARCB1, and SMARCA4. Frequency of mutations except SNV is given in %/IVMs analyzed. IVMs with detected LOH of chr 1p or 22q are depicted in grey

Next, we focused on the most common genetic alterations reported so far in meningiomas, including the NF2, SMARCB1, as well as TRAF7, AKT1, SMO, KLF4, PIK3CA, and TERT in non-NF2 mutated meningiomas (Fig. 3) [51]. NF2 was the most frequently affected gene in 44% of the tumor samples (*n* = 8/18). In detail, in four cases the NF2 gene was disrupted by frameshift mutations (*n* = 4/18, 22%), in three cases we discovered a stop-gain mutation (*n* = 3/18, 17%) and in one case a mutation causing a splicing error of the NF2 gene was detected (*n* = 1/18, 6%) (Table 3). Notably, 7 out of 8 female patients harbored NF2 mutations. According to the literature, TRAF7 is the most often affected gene in non-NF2 mutated meningiomas [1, 9]. In our series of IVMs, we failed to identify any alterations in the TRAF7 gene. Only few synonymous single nucleotide variations (SNV) were detected which were already known polymorphisms. Interestingly, two SMO mutations (R168H and P698R) were identified in our set of IVMs, but they differed from the known activating SMO mutations L412F and W535L previously described in olfactory groove meningiomas [1, 9]. Krueppel-like factor 4 (KLF4) mutations (K409Q) have a high prevalence in secretory meningiomas but were not observed in our IVM cohort [43]. Furthermore, the AKT1 gene was altered in one patient only; however, the discovered 3′-UTR mutation (rs17846826) has unknown clinical significance. Finally, only one TERT mutation (A279T) was detected in one WHO°I tumor, which differs from the previously reported TERT alterations in meningiomas (C228T and C250T). The low-incidence SMARCB1 R377H mutation was detected in one WHO°II IVM and its corresponding tumor recurrence. In the other WHO°II IVM, a SMARCA4 (G1644S) mutation was detected. Taken together, NF2 mutation was the most frequent event in IVMs, whereas other common genetic alterations reported in the literature were not detected. However, aggressive IVMs harbor SMARC mutations indicating the involvement of the SWI/SNF complex genes in the pathogenesis of aggressive IVMs.

**Table 3:** In silico predictions of possible damaging mutations

Gene	Nucleotide Substitution	Exon	Amino Acid change	Type of Mutation
*APC*	G5827A	14	V1943I	missense
	G5881A	16/17	V1961I	missense
*GABRA6*	G805A	7	V269I	missense
*GSE1*	C2171T	10	S724L	missense
	C2264T	10	S755L	missense
	C2483T	11	S828L	missense
*KDR*	G2443C	17	E815Q	missense
*SMO*	G503A	2	R168H	missense

### Novel mutations in intraventricular meningiomas

**Fig. 4:**
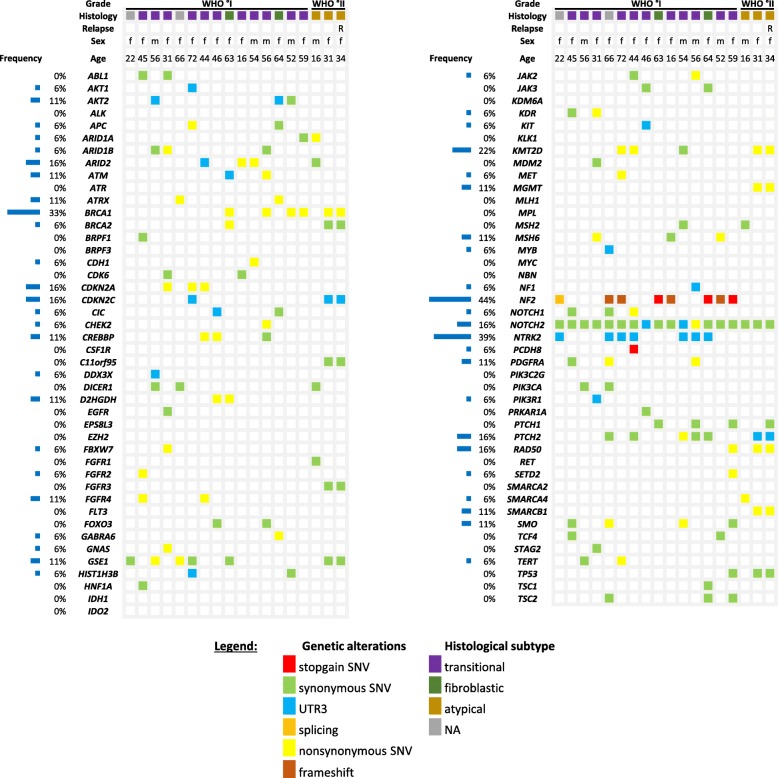
Gene panel. Tabularly overview of all investigated genetic alterations covered by the targeted sequencing panel. Frequency of occurrence is given in %/IVMs analyzed (*n* = 18)

Subsequently, we analyzed our targeted gene panel for other frequent genetic alterations (Fig. 4). Apart from NF2, the most affected genes in our IVM cohort were NTRK2 (39%, *n* = 7/18), BRCA1 (33%, *n* = 6/18), KMT2D (22%, *n* = 4/18), CDKN2A (18%, *n* = 3/18), and CDKN2C (18%, n = 3/18). NTRK2 (neurotropic receptor tyrosine kinase 2) encodes a neurotrophin receptor, which is known to play a role in gliomagenesis [8, 29]. However, we only detected untranslated region (UTR) mutations in seven WHO°I IVMs with unknown clinical significance. BRCA1, a well-known breast cancer tumor suppressor, was altered in 4 WHO°I IVMs and in one WHO°II and its recurrent tumor (n = 6/18). The identified missense mutations suggested to be most likely benign according to ClinVar [10]. KMT2D plays a critical role in regulating development, differentiation, metabolism, and tumor suppression [17]. We found four different mutations in the KTM2D gene in three WHO°I IVMs (P2390L, M3870I, and P4620L) and in one WHO°II IVM (E1833G) and its corresponding tumor recurrence, whereof only one mutation (M3870I) has been previously reported with a most likely benign clinical course. The common missense variant at CDKN2A (rs3731249) was found in two WHO°I IVM samples, which has been shown to be associated with increased susceptibility to acute lymphoblastic leukemia [58]. However, the nonsynonymous mutation CDKN2A A79V and three UTR mutations in the CDKN2C gene (rs41285700; rs41285702) were not reported previously.

### In silico prediction of possible pathogenic genes

By using in silico algorithms including SIFT, PhyloP, PolyPhen2, LIB-LRT, and MutationTaster, we were able to identify five possible pathogenic mutations: APC, GABRA6, GSE1, KDR, and SMO (Table 3). In one NF2-mutated IVM WHO°I, we detected two missense mutations (V1943I and V1961I) in the Adenomatous polyposis coli (APC) gene, which is a crucial tumor suppressor in the WNT/b-Catenin pathway. The GABRA6 (Gamma-Aminobutyric Acid Type A Receptor Alpha6 Subunit) mutation V269I (*n* = 1 WHO°I IVM) is associated with epilepsy, but, until now, has not been linked to cancer [22]. GSE1 (genetic suppressor element 1), a proline-rich protein, also known as KIAA0182, was found in two WHO°I IVMs and has been shown to possess oncogenic properties in human breast cancer cells, gastric cancer and in neuroepithelial stem cells [7, 14, 24]. Finally, regarding KDR, kinase insert domain receptor, we found one missense mutation E815Q in one WHO°I IVM, which has not been reported previously.

## Discussion

Despite growing interest in the molecular pathogenesis of meningiomas, intraventricular meningiomas have not been investigated on a molecular level yet. They comprise a rare subset of meningiomas and remain challenging in the clinical setting due to their delicate location and their unhindered growth within the ventricles. In this study, we analyzed our institutional cohort of 25 patients with IVMs which is among the largest published IVM cohorts to date [19]. To unravel the underlying genetic alterations, we performed targeted panel sequencing of 18 IVMs including one matching recurrent tumor. We discovered loss of chr 22q and 1p in 89 and 44% of the cases, respectively. NF2 mutations were found in 44% of the cases, while common genetic alterations in meningiomas of other locations (TRAF7, AKT1, SMO, KLF4, PIK3CA, and TERT) were lacking in non-NF2-associated cases. Interestingly, we found the low-incidence SMARCB1 and SMARCA4 mutations in WHO°II IVMs. Furthermore, two SMO mutations and one TERT mutation different from the ones previously described were detected. Regarding possible alternative pathogenic genes covered by the panel, we detected APC mutation and in silico predicted pathogenic mutations of GABRA6, GSE1, KDR.

Patient demographics, presenting symptoms, tumor location, WHO grade and histological subtypes of our patient cohort matched with the findings of a recent systematic review that summarized all 681 IVM cases published so far [39]. In eight patients (36.4%), intraventricular meningiomas were incidental findings which is in accordance with the literature for incidental findings of intracranial meningiomas of all locations (38–42%) [27, 55]. In this analysis, out of 494 cases with outcome data available, 26 (5.3%) tumors relapsed within a mean interval of 26 months [39]. In our institutional cohort, median PFS was 79 months and PFS at 5 years was 86%.

Copy number profiles were calculated from the low-resolution the sequencing array data. This method was previously described and showed high concordance compared to 450 k methylation data, but may differ from high-resolution techniques such as array comparative genomic hybridization [47, 50]. Evaluation of the CNV plots revealed chromosomal losses at chr 22q in 89% of the samples. At this chromosomal arm, the critical tumor suppressor NF2 is located. LOH at chromosome 22q is an early event in the tumorigenesis of meningiomas and occurs with a frequency of 40–80% [28, 31, 34]. The second most prevalent chromosomal alteration in our cohort was the loss of chr 1p in 44% of the samples. According to the literature, partial or complete loss of chromosome 1p is the second most frequent chromosomal abnormality found in meningiomas and is associated with more aggressive and recurrent meningiomas while rarely occurring in WHO°I meningiomas [4, 18, 31, 33]. In our cohort, chromosomal loss of 1p was detected in all WHO°II IVMs (*n* = 2 WHO°II, *n* = 1 recurrent WHO°II tumor), but also in 33% (*n* = 5/15) of WHO°I IVMs.

Next, we focused on the most prevalent genetic alterations reported in meningiomas so far, including NF2, SMARCB1, as well as TRAF7, AKT1, SMO, KLF4, PIK3CA, and TERT in non-NF2 mutated meningiomas [1, 9, 20]. NF2 was affected in 44% of our tumor samples (*n* = 8/18) by frameshift mutations (*n* = 4/18), stop-gain mutations (*n* = 3/18) and splicing error (n = 1/18), which is in accordance with the literature as the primary inactivated tumor suppressor in meningiomas [6, 9, 28]. Interestingly, 7 out of 8 female patients (87%) harbored NF2 mutations which is in line with the largest dataset available which reported on 112 NF2-mutated meningiomas including 79 female patients corresponding to a female preponderance of approximately 70% [9]. The tumor suppressor TRAF7, which exclusively occurs in non-NF2 meningiomas, is the second most altered gene with mutations occurring in approximately 25% of cases [1, 9]. However, in our IVM cohort, we failed to identify any alterations in the TRAF7 gene. KLF4 K409Q mutations were exclusively found in the presence of TRAF7 mutations and are commonly associated with secretory meningiomas [9, 43]. Due to lack of secretory meningiomas and TRAF7 mutations in our cohort, the absence of KLF4 K409Q in our cohort may be expected. SMO activating mutations (L412F and W535L) have been previously identified with a predilection of olfactory groove meningiomas [1, 9]. In our cohort, two WHO°I IVMs displayed two distinct SMO mutations (R168H and P698R) which differ from the ones already known. The location of the SMO R168H missense mutation is highly conserved among the human, mouse, and drosophila proteins, and is positioned adjacent to a cystine residue, so the R168H change may influence protein structure, function, and hedgehog signaling [54]. The SMO P698R mutation is predicted by in silico analysis as pathogenic but to date functional data for this mutation is lacking. Finally, the TERT A279T mutation was found in our IVM cohort, which deviates from the previously reported TERT mutations in meningiomas (C228T and C250T) [46]. Functional data in esophageal cancer cells overexpressing TERT A279T induced telomere dysfunction but interestingly decreased proliferation of these cells [57]. In one WHO°II and its corresponding recurrent tumor, the already known driver mutation SMARCB1 R377H was detected [6, 52]. Moreover, in the second WHO°II IVM, we detected a SMARCA4 missense mutation G1644S. SWI/SNF complexes play a critical role in coordinating chromatin architecture and gene expression. They alter the structure of reconstituted chromatin particles in an ATP-dependent manner and make chromatin more accessible for transcription factor binding [44]. This is of particular interest since several families with multiple meningiomas and schwannomas harbor germline mutations in the SWI/SNF core complex unit SMARCB1. Less frequent, sporadic mutations occur in WHO°I and WHO°II meningiomas concurrently with NF2 mutations and are associated with poorer prognosis [52]. Moreover, mutation in SMARCB1 are typically found in rhabdoid tumors and epithelioid sarcomas [44]. Even more rarely, a SMARCA4 mutation has been reported in one anaplastic meningioma [11]. Taken together, members of the SWI/SNF complex may play a role in the pathogenesis of aggressive IVMs [11, 44].

A number of studies highlight the importance of the Wnt signaling pathway in meningioma [38]. APC plays a major role as a tumor suppressor by forming the beta-catenin destruction complex together with AXIN and GSK-3b. Gross deletion was detected in approximately half of meningiomas [37]. The two missense mutations (V1943I and V1961I) found in one WHO°I IVM were not described previously but according to our in silico analysis may be damaging and might therefore increase the Wnt signaling. Furthermore, we discovered in silico predicted pathogenic mutations of GABRA6, GSE1 and KDR. The GABRA6 mutation is associated with childhood absence epilepsy but, to date, has not been linked to cancer [22]. GSE1, also known as KIAA0182, has been shown to possess oncogenic properties in human breast cancer cells and gastric cancer and accelerates tumorigenesis in neuroepithelial stem cells of Gorlin syndrome patients, who are predisposed to medulloblastoma due to PTCH1 mutation [7, 14, 24]. One KDR missense mutation E815Q was found in one WHO°I IVM, which has not been reported previously. KDR, also known as VEGFR2, plays an important role in angiogenesis which is essential for the growth of any solid tumor [40]. KDR mRNA and protein expression levels have been investigated several times in meningiomas and found contradicting results [23, 40, 42]. Nevertheless, activating mutations of KDR (D717V and A1056T) have been discovered in angiosarcomas and have been verified in vitro [3]. Up to now, no data for the missense mutation E815Q is available. Taken together, APC, KDR and GSE1 mutations might contribute to meningioma growth, however, validation by Sanger sequencing and functional analyses are needed to strengthen these preliminary findings.

## Conclusion

In conclusion, NF2 mutations were the most frequent genetic alteration in our cohort of IVMs, whereas other common genetic alterations previously reported in WHO°I meningiomas of distinct tumor locations were not found. However, members of the chromatin remodeling complex SWI/SNF SMARCB1 and SMARCA4 may play a role in the pathogenesis of aggressive IVMs.

## Additional file

Additional file 1: Sequencing data of IVMs. (XLSX 1955 kb)

## Data Availability

The data generated or analyzed during this study are included in this published article and its Additional file 1.

## Abbreviations

Chr: Chromosome
CNV: Copy number variation
EOR: Extent of resection
GTR: Gross total resection
IVM: Intraventricular meningioma
LOH: Loss of heterozygosity
MRI: Magnetic Resonance Imaging
PFS: Progression-free survival
SNV: Single nucleotide variant
STR: Subtotal resection
WHO: World Health Organization
